# Prediction of antiviral efficacy in patients with chronic hepatitis C by changes in forkhead box protein 3 levels

**DOI:** 10.3892/etm.2014.1675

**Published:** 2014-04-08

**Authors:** JIA-BAO CHANG, RONG XUE, ZHEN-XIAN ZHOU, YAN-HONG FENG, WEI-WEI DAI, JIE QIU, YONG-FENG YANG

**Affiliations:** Department of Liver Disease, The Second Affiliated Hospital, Southeast University, Nanjing, Jiangsu 210007, P.R. China

**Keywords:** chronic hepatitis C, CD4^+^CD25^+^ forkhead box P3^+^ regulatory T cells, hepatitis C virus-RNA load

## Abstract

The aim of the present study was to investigate the distribution of CD4^+^CD25^+^ regulatory T cells (Tregs) in the peripheral blood of patients with chronic hepatitis C; in addition to identifying whether the distribution of CD4^+^CD25^+^ Tregs predicts the efficacy of antiviral therapy for HCV. The expression of CD4^+^CD25^+^ forkhead box protein (FOXP) 3^+^ Tregs within a CD4^+^ T cell population was detected in the peripheral blood obtained from patients with chronic hepatitis C and from healthy control subjects using flow cytometry. The hepatitis C virus (HCV)-RNA load was measured using quantitative-fluorescence polymerase chain reaction. CD4^+^CD25^+^FOXP3^+^ Tregs accounted for 14.24±1.33% of the CD4^+^ T cells in the peripheral blood of patients with chronic hepatitis C, which was higher than that of the healthy control subjects (5.62±1.21%; P<0.001). Furthermore, the frequency of CD4^+^CD25^+^FOXP3^+^ Tregs in CD4^+^ T cells of the peripheral blood positively correlated with the HCV-RNA load (r=0.73; P=0.032). Therefore, the results of the present study indicated that the expression of CD4^+^CD25^+^FOXP3^+^ Tregs increased in patients that were chronically infected with HCV and positively correlated with the HCV-RNA load.

## Introduction

Hepatitis C is a disease that infects ~200 million individuals worldwide (1). In 2004, there were 220,000 patient mortalities from hepatitis C in China. A significant clinical characteristic of the hepatitis C virus (HCV) infection is that >80% of patients with the acute infection ultimately develop a chronic persistent infection. Chronic hepatitis C is a significant causative factor of cirrhosis and primary hepatocellular carcinoma. However, the mechanism underlying chronic HCV infection remains unclear. Virus variation, specifically gene mutation in the hypervariable region, may correlate with chronic HCV infection. Cabrera *et al* (2) investigated the quantity and phenotype of CD4^+^CD25^+^ regulatory T cells (Tregs) in the peripheral blood and their effects on the HCV-specific cellular immune response in patients with chronic HCV infection and in healthy control subjects. The number of forkhead box protein (FOXP) 3^+^ Tregs in the peripheral blood was identified to be greater in patients with chronic HCV infection when compared with the healthy control subjects. An *in vitro* study demonstrated that the removal of FOXP3^+^ Tregs from the peripheral blood, obtained from infected patients, enhanced the CD8^+^ T cell-mediated antigen-specific response (3). The number of Tregs was markedly higher in patients with chronic HCV infection when compared with the recovered patients and healthy control subjects. The phenotype of Tregs in the three groups was approximately the same, which excluded the hypothesis that CD4^+^CD25^+^ Tregs were capable of strengthening the proliferation of specific T cells and the production of interferon (IFN)-γ in HCV-infected patients. Furthermore, the number of CD4^+^CD25^+^ Tregs was shown to positively correlate with the HCV-RNA content and negatively correlate with the degree of liver inflammatory activity. Boettler *et al* (4) identified that persistent HCV infection elevated the number of CD4^+^CD25^+^ Tregs and directly suppressed the activity of HCV-specific CD8^+^ T cells. Tajimi *et al* (5) further confirmed that chronic HCV infection induced the production of HCV core antigen and nonstructural protein 3 antigen-specific Tregs, which aggravated the HCV infection.

Chronic HCV infection in the human liver may result in serious liver damage and require liver transplantation. Liver biopsy following transplantation has revealed that the degree of inflammatory injury within the transplant tissue negatively correlates with the number of FOXP3^+^ Tregs in the peripheral blood (2). At present, few clinical investigations have been conducted regarding the prediction of the antiviral effect on chronic hepatitis C. High cost and the long treatment cycle associated with IFN therapy are factors that are restricting patients from undergoing therapy. Thus, the ability to predict antiviral efficacy is likely to have a positive impact on clinical investigations. The serum level of FOXP3 reflects the activity of CD4^+^CD25^+^ Tregs and the condition of immune function in patients, which aids in predicting the efficacy of antiviral treatment.

The present study involved Han Chinese patients from Jiangsu and Anhui that exhibited a negative virus index and abnormal liver function. Patients with chronic hepatitis C were selected to investigate the immunomodulatory effect of Tregs and FOXP3-related genes on chronic hepatitis C liver disease. In addition, the correlation between FOXP3 and clinical symptoms was analyzed and data exhibiting unique Chinese characteristics and independent intellectual property rights was obtained. The aim was to provide data with important clinical value on treatment efficacy and prognosis, and further clarify the pathogenesis of chronic HCV infection.

## Materials and methods

### Study subjects

A total of 120 Han Chinese patients exhibiting chronic HCV infection that had been admitted to the Second Affiliated Hospital (Nanjing, China) between 2008 and 2010 and 102 healthy control subjects were enrolled in the study. The present study was conducted in accordance with the Declaration of Helsinki and with approval from the Ethics Committee of the Second Affiliated Hospital. Written informed consent was obtained from all the participants. The patients were from Jiangsu and Anhui and exhibited positive anti-HCV, negative hepatitis virus indicators and abnormal liver function. The patients had not undergone antivirus or immune-regulation therapy within the past six months. The healthy control subjects did not suffer from any disease, including autoimmune disease or diabetes. Written informed consent was obtained from the patients.

### Immunohistochemistry assay

Venous blood samples (3 ml) were collected from each patient, from which the serum was isolated and divided into two aliquots; one was used for the detection of biochemical indicators and the other was stored at −20°C for the detection of various virus antibodies. Chemiluminescence immunoassays were performed to detect the presence of tumor markers, including α-fetoprotein.

### FOXP3 gene expression

*Taq*Man-fluorescence quantitative polymerase chain reaction (qPCR; Applied Biosystems, Carlsbad, CA, USA) was employed to analyze gene expression. Specific PCR primers and fluorescence labeled *Taq*Man probes were designed according to the gene sequences of human FOXP3. The standard was established through a gene clone to conduct reverse transcription PCR amplification. Quantitative determination of gene expression was performed using a fluorescence qPCR instrument.

### Cell culture

Peripheral blood was collected from patients with chronic HCV infection and was combined with EDTA for anticoagulation. This was followed by plasma separation and centrifugation. The buffy coat cells were collected and combined with an equal volume of physiological saline and lymphocyte separation medium. Mononuclear cells were isolated by density gradient centrifugation over Ficoll-Paque (Amersham Pharmacia Biotech, Uppsala, Sweden) and labeled with fluorescein isothiocyanate conjugated anti-CD4 and R-phycoerythrin conjugated anti-CD25 antibodies; this was followed by analysis of the CD4^+^CD25^+^ T cells. The CD4^+^CD25^+^ T cells for culturing were separated and collected by flow cytometry (Beckman Coulter, Inc., Brea, CA, USA) or immunomagnetic separation.

### FOXP3 gene mutation

Specific primers and oligonucleotide probes were designed. PCR was conducted with a sequence-specific oligonucleotide probe or a sequenator.

### Gene polymorphism

Using an anticoagulant, DNA was extracted from the peripheral blood of the patients. The primers were designed according to the gene sequences of the cytokines, including FOXP3 and the non-coding sequences of their promoters and enhancers, which were registered in the GenBank database (National Center for Biotechnology Information, Bathesda, MD, USA). PCR was conducted for amplification, and enzyme digestion, electrophoresis and sequencing were performed for gene detection.

### Statistical analysis

The statistical analysis of T cells and cytokines was performed using the paired t-test. Comparison of the mean values of each group was conducted using the rank-sum test and comparison of enumeration data was performed with the χ^2^ or U test. Analysis of the correlation between susceptibility genes and the disease was conducted via comparison of gene frequency using the χ^2^ test (SPSS software; SPSS, Inc., Chicago, IL, USA). Relative risk (RR) was calculated according to Woolf’s formula and RR factors were analyzed using multivariate logistic regression.

## Results

### CD4^+^CD25^+^FOXP3^+^ Tregs frequency

CD4^+^CD25^+^FOXP3^+^ Tregs accounted for 14.24±1.33% of CD4^+^ T cells in the peripheral blood obtained from patients with chronic hepatitis C, which was higher than that of the healthy control subjects (5.62±1.21%; P<0.001, Table I).

### Correlation assay

The HCV-RNA viral load was transformed into a natural logarithm and its linear correlation with the frequency of CD4^+^CD25^+^FOXP3^+^ Tregs within a CD4^+^ T cell population was analyzed (Table II). The frequency of CD4^+^CD25^+^FOXP3^+^ Tregs in the CD4^+^ T cells of the peripheral blood positively correlated with the HCV-RNA viral load (r=0.73; P=0.032).

## Discussion

Previous studies identified a type of suppressor T cell in patients with chronic infection that specifically expressed the α-chain of interleukin (IL)-2, termed CD25. These cells are defined as CD4^+^CD25^+^ Tregs and predominantly secrete IL-10 and transforming growth factor-β, and express the transcription factor, FOXP3 (6). Activated CD4^+^CD25^+^ Tregs are able to inhibit the proliferation, differentiation and cytokine secretion of CD4^+^ and CD8^+^ T cells in a cellular contact-dependent manner (7). A number of studies have demonstrated that the percentage of CD4^+^CD25^+^ Tregs is significantly greater in patients with chronic HCV infections when compared with recovered patients (7.3 vs. 2.5%; P=0.002). *In vitro* studies have identified that CD4^+^CD25^+^ Tregs suppress the proliferation and function of HCV-specific CD8^+^ T cells (7). CD4^+^CD25^+^ Tregs, activated by the T cell receptor, exhibit strong inhibitory effects on the proliferation of antigen specific CD4^+^ and CD8^+^ T cells and the formation of T memory (Tm) cells. These cells have also been shown to promote apoptosis of CD8^+^ Tm cells (3,8). In addition, when compared with patients exhibiting a single infection, the prognosis was worse for HCV infected patients experiencing additional infections, whose Tm level was relatively low.

FOXPs, characterized by a fork head structure, are a family of transcription factors that perform various functions in the process of cell development. FOXP3 is closely associated with the development and biological function of Tregs and downregulates the immune response. FOXP3 is a transcription factor that is expressed by CD4^+^CD25^+^ Tregs and is the predominant control gene for the development and function of CD4^+^CD25^+^ Tregs. Gene mutation of FOXP3 can result in the deficiency or dysfunction of CD4^+^CD25^+^ Tregs and lead to autoimmune and inflammatory disorders. Furthermore, FOXP3^+^ Tregs are a subtype of T cells that express CD4, CD25 and FOXP3, and are essential to the dynamic regulation of human immune homeostasis (9). The development and dysfunction of Tregs is closely associated with a variety of immune-related diseases, including autoimmune disease, inflammatory response, acute and chronic communicable diseases, immune tolerance to tumors, transplant rejection and the physiological changes of allergic disease (10).

Tregs are derived from the thymus and account for 5–10% of CD4^+^ T cells in the peripheral blood of healthy mice and humans. Tregs are capable of suppressing the amplification of other T cells. However, the functional mechanism of Tregs remains unclear. Although Treg function closely correlates with cytotoxic T-lymphocyte antigen 4 (CTLA-4), glucocorticoid-induced TNFR family related gene (GITR), transforming growth factor-p and IL-10, these are not specifically expressed by Tregs. CTLA-4 and GITR are associated with Treg activation and are expressed on the surface of other activated CD4^+^ T cells (10). Hori *et al* (11) identified that FOXP3 is specifically expressed by Tregs and is essential to the development and biological function of Tregs. Furthermore, the authors demonstrated that CD4^+^CD25^+^ Tregs with FOXP3 transduction inhibited the proliferation of lymphocytes and the occurrence of inflammatory bowel disease and gastritis in mice. In addition, *in vitro* experiments have demonstrated that CD4^+^CD25^+^ Tregs restrain the proliferation of naïve T cells and the production of their cytokines, and upregulate the expression of surface molecules that are associated with the immune modulating function of natural Tregs. Recombination-activating gene 2-deficient mice exhibited no detectable immune-pathological changes following *Pneumocystis carinii* infection, which was controlled by adoptive infusion of CD4^+^CD25^+^ T cells into the mice; however, it resulted in severe lung tissue injury, which was alleviated following the injection of FOXP3^+^ Tregs (11). In mice with *Candida albicans* infection, a reduction in the FOXP3^+^ Treg level effectively controlled the infection, however, simultaneously enhanced the pathological injury that was induced by the gastrointestinal inflammation (12). Chronic HCV infection of the human liver may result in serious liver damage and require liver transplantation. Liver biopsies conducted following transplantation have revealed that the degree of inflammatory injury within the transplant tissue negatively correlates with the number of FOXP3^+^ Tregs in the peripheral blood (2).

A previous study indicated that FOXP3^+^ Tregs were capable of regulating the intensity of the pathological response mediated by immune effector cells. In addition, the study showed that the predominant function of Tregs was to respond to tissue injury-related signals, thus, reducing tissue damage (13). The present study demonstrated that the percentage of CD4^+^CD25^+^FOXP3^+^ Tregs in CD4^+^ T cells obtained from the peripheral blood was greater in patients with chronic hepatitis C than in the healthy control subjects, indicating that Tregs are overexpressed in HCV infected patients. In addition, the results indicated that CD4^+^CD25^+^FOXP3^+^ Tregs may be involved in the process of HCV infection and strengthen the inhibitory effect on the proliferation of virus-specific CD8^+^ T cells. The percentage of CD4^+^CD25^+^FOXP3^+^ Tregs was greater in patients with chronic hepatitis C than in the healthy control subjects and CD4^+^CD25^+^FOXP3^+^ Tregs were able to inhibit the response of CD8^+^ T lymphocytes to various virus antigens, thus, promoting chronic HCV infection. Patients with chronic hepatitis C may produce certain factors that stimulate the hyperplasia and maturity of CD4^+^CD25^+^FOXP3^+^ Tregs.

Effective control of HCV infection requires the synergistic action of CD4^+^ Th1 cells and cytotoxic T cells (CTL). CTLs are not able to differentiate if CD4^+^ Th1 cells are deficient or cease to perform their activation and proliferation functions, ultimately resulting in chronic viral infection. Previous studies have demonstrated that chronic pathogen infection induces an upregulation of the expression of CD4^+^CD25^+^ Tregs within the host and inhibits the response of antigen-specific CD4^+^ T cells. In addition, the removal of CD4^+^CD25^+^ Tregs significantly reduces the pathogen infection rate (14). Accumulative experimental evidence has identified that the level and persistence of FOXP3 expression are important for the maturation and function of Tregs (15,16). In addition, the combination of FOXP3 with multiple transcription factors and transcription complexes with enzymatic activity, is essential to suppress the transcriptional activation of cytokines in T cells. Post-translational modification and transcription complex assembly of the FOXP3 protein, as well as its modification of enzyme activity, are dynamically regulated via signals from the T cell and cytokine receptors (17). Thus, human and murine FOXP3^+^ Tregs exhibit marked differences in development, function and plasticity. This provides novel areas for investigation, and challenges for transforming basic immunological studies into clinical research, including cell therapy based on Tregs and the understanding of predominant infectious diseases which are unique to humans. The present study demonstrated that the HCV-RNA load positively correlated with the expression of CD4^+^CD25^+^FOXP3^+^ Tregs, which was consistent with the results of a previous studies (18).

In conclusion, the present study indicates that CD4^+^CD25^+^ Tregs are significant in the initiation of chronic HCV infection. The transcription factor, FOXP3, was capable of regulating and inducing the proliferation of CD4^+^CD25^+^ Tregs in the peripheral blood obtained from HCV infected patients. In addition, FOXP3 increases the inhibitory effect of Tregs on the immune system and promotes an incomplete immune response to the virus, ultimately resulting in a chronic viral infection.

## Figures and Tables

**Table I:** Frequency of CD4^+^ CD25^+^ FOXP3^+^ Tregs observed in CD4^+^ T cells obtained from the peripheral blood.

Group	Patients (n)	Frequency of CD4^+^CD25^+^FOXP3^+^ Tregs (%)
Chronic HCV	120	14.24±1.33*
Healthy control	102	5.62±1.21

* P<0.001.

FOXP3, forkhead box protein 3; Tregs, regulatory T cells; HCV, hepatitis C virus.

**Table II:** Correlation between the expression level of CD4^+^CD25^+^FOXP3^+^ Tregs and the HCV-RNA viral load.

Patients (n)	HCV-RNA viral load	Frequency of CD4^+^CD25^+^FOXP3^+^ Tregs (%)*
38	<5	5.26±1.82
67	5–7	9.31±1.24
53	7–9	12.57±1.14
64	>9	14.68±2.03

* r=0.73; P=0.032;

FOXP3, forkhead box protein 3; Tregs, regulatory T cells; HCV, hepatitis C virus.
